# Genetic and molecular factors in determining grain number per panicle of rice

**DOI:** 10.3389/fpls.2022.964246

**Published:** 2022-08-04

**Authors:** Yue Lu, Mingli Chuan, Hanyao Wang, Rujia Chen, Tianyun Tao, Yong Zhou, Yang Xu, Pengcheng Li, Youli Yao, Chenwu Xu, Zefeng Yang

**Affiliations:** ^1^Key Laboratory of Plant Functional Genomics of the Ministry of Education, Jiangsu Key Laboratory of Crop Genomics and Molecular Breeding, College of Agriculture, Yangzhou University, Yangzhou, China; ^2^Jiangsu Key Laboratory of Crop Genetics and Physiology, Jiangsu Co-Innovation Center for Modern Production Technology of Grain Crops, Yangzhou University, Yangzhou, China; ^3^Joint International Research Laboratory of Agriculture and Agri-Product Safety of the Ministry of Education, Yangzhou University, Yangzhou, China

**Keywords:** grain number, panicle architecture, branch differentiation, vascular development, rice

## Abstract

It was suggested that the most effective way to improve rice grain yield is to increase the grain number per panicle (GN) through the breeding practice in recent decades. GN is a representative quantitative trait affected by multiple genetic and environmental factors. Understanding the mechanisms controlling GN has become an important research field in rice biotechnology and breeding. The regulation of rice GN is coordinately controlled by panicle architecture and branch differentiation, and many GN-associated genes showed pleiotropic effect in regulating tillering, grain size, flowering time, and other domestication-related traits. It is also revealed that GN determination is closely related to vascular development and the metabolism of some phytohormones. In this review, we summarize the recent findings in rice GN determination and discuss the genetic and molecular mechanisms of GN regulators.

## Introduction

Rice (*Oryza sativa* L.), one of the most widely consumed food crops, feeds over a half of world population, and provides more than 21% of the dietary calories worldwide (Fitzgerald et al., 2009; Muthayya et al., 2014; Ito, 2019). The total milled rice consumption is 490 million tons in 2018, and is predicted to be 590 million tons in the year 2040 (Ito, 2019). Therefore, increasing the rice grain yield is an essential task for the fulfillment of global food security.

Rice grain yield is a complex quantitative trait determined by three major yield components, panicle number (PN), grain number per panicle (GN), and grain weight (GW; Xing and Zhang, 2010; Zuo and Li, 2014). Among them, GN is suggested to be the critical trait in increasing grain yield in the breeding practice (Huo et al., 2017; Gouda et al., 2020b; Wu et al., 2021). According to the ideal plant architecture model, low tillering and large panicle (200–250 grains per panicle) are the future targets for the breeding of rice (Khush, 2005). In a survey of yield traits covering 200 *japonica* rice cultivars bred in central China, a significant increase can be observed for GN during the past 30 years, and many GN-associated genes experienced artificial selection during the breeding process (Xiao et al., 2021), suggestive potential pivot of GN in genetic improvement of rice yield.

GN is mainly determined by the panicle architecture and branch differentiation, which are closely associated with the phytohormone pathways and vascular development (Terao et al., 2010; Duan et al., 2019; Deveshwar et al., 2020). It has been shown that several important genes associated with these processes have significant potential in improving GN and rice grain yield. *Gn1a* (*Grain number 1a*)/*CKX2* (*Cytokinin oxidase 2*) is the first isolated GN-associated gene, which was identified through map-based cloning (Ashikari et al., 2005). Favorable allele of *Gn1a* increased rice grain yield by up to 11.9% when introgressed into an elite *japonica* rice cultivar Kongyu 131 (Feng et al., 2017), suggestive great potential of GN-associated genes in the breeding practice of rice yield improvement. Another GN-associated QTL, *GN4-1*, caused a 14.3% increase of grain yield when introgressed into an elite *indica* cultivar (Zhou et al., 2018). Similarly, introduction of a GN-associated gene *NOG1* (*Number of Grains 1*) into a deficient rice cultivar increased the grain yield by 25.8% (Huo et al., 2017). Moreover, a major quantitative trait loci (QTL) of GN, *qPE9-1* (*QTL panicle erect 9–1*)/*DEP1* (*Dense and Erect Panicle 1*), which control the dense and erect panicle architecture, has been applied in breeding since the 1980s, and has occupied a dominant position among *japonica* rice cultivars in northern China, long before the gene was isolated (Yan et al., 2007). It can be inferred that the beneficial alleles of GN-associated genes or QTLs confer high-yield potential in rice breeding. Identification and mechanic studies of genes associated with GN would provide valuable gene resources for rice yield improvement. In this review, we summarized current progress on the molecular and genetic basis under the control of GN, and raise future perspectives on the approaches for improving GN in rice breeding.

### Genetic characteristics and influencing factors of GN in rice

The rice panicle consists of main rachis, rachis branches (including primary and secondary branches) and spikelets. Spikelets are the basic units of the inflorescence, which are attached to the branches through pedicels, while branches were arise from the nodes of main rachis (Wang and Li, 2005; Xing and Zhang, 2010). As a canonical quantitative trait, GN is controlled by multiple genes, and can be affected by various environmental factors (Yin et al., 2021; Li et al., 2022). The broad-sense heritability of GN is relatively high, which ranged from about 70 to 90% in different studies (Tuhina-Khatun et al., 2015; Roy and Shil, 2020), suggesting that genetic factors are the major determinant of GN. Furthermore, GN is positively correlated with panicle length, number of primary and secondary branches (Rebolledo et al., 2016; Li et al., 2021b).

The spikelet fertility is influenced by environmental conditions, such as temperature, nutrition and water supply. Hence, the environment conditions and cultivation methods also have significant effect on GN. High temperatures occurring at flowering and young microspore stages cause decrease of GN in rice due to spikelet degeneration and decreased pollen viability (Hu et al., 2021; Park et al., 2021); while chilling stress also results in reduced GN through impairing pollen germination and reducing spikelet fertility (Zeng et al., 2017; Hussain et al., 2018; Ali et al., 2021). Water stress during the meiosis stage leads to a severe reduction (40–45%) in GN due to pre-flowering spikelet abortion (Kato et al., 2008; Yang et al., 2019). Cultivation methods also affect GN through regulating the light and nutrition availability. Evidence revealed that high density planting causes decreased GN due to limited light and nutrition supply, while application of nitrogen fertilizer has a positive effect on GN (Jiang et al., 2021; Ju et al., 2021; Wang et al., 2022b). Both the genetic background and the cultivation and environmental influences are essential for the determination of GN.

### Regulation of GN by panicle architecture

The panicle architecture, including panicle length, shape, and the number and arrangement of primary and secondary branches, is an important factor for determining GN. G-protein signal pathway is widely involved in controlling plant growth and morphogenesis (Perfus-Barbeoch et al., 2004; Ghusinga et al., 2022). *qPE9-1* (*QTL panicle erect 9–1*)/*DEP1* (*Dense and Erect Panicle 1*) is characterized as a major QTL for rice grain yield through regulating panicle architecture. *qPE9-1*/*DEP1* encodes the γ subunit of a G-protein, and a deletion mutation of the cysteine-rich domain enhanced the transmission of G-protein signaling, which leads to an erect and compact panicle shape, shortened panicle length, enhanced cell division and increased number of branches (Huang et al., 2009b; Zhou et al., 2009; Xu et al., 2016). The favorable allele of *qPE9-1/DEP1* has been widely applied in the practice of rice breeding for high yield in China (Sun et al., 2014; Liu et al., 2018).

In addition to the G-protein signal pathway, many other genes were associated with the panicle architecture regulation. The *SP1* (*Short Panicle 1*) gene encodes a peptide transporter family protein, which is predominantly expressed in the branch vascular bundle. Enhanced expression of *SP1* in young panicles can promote the elongation of the cob, increase the panicle size, and increase GN (Li et al., 2009). The *sped1-D* gene, encoding a pentatricopeptide repeat protein, affects panicle structure through blocking the action of *GID1L2* (*GA-INSENSITIVE DWARF1-L2*), *RFL* (*RICE FLORICAULA*) and *WOX3* (*WUSCHEL-related homeobox 3*), and caused shortened pedicels and decreased GN (Jiang et al., 2014). Additionally, mutation of *DEP3* (Qiao et al., 2011) and *EP3* (*Erect Panicle 3*; Piao et al., 2009) were also associated with erect panicle shape, condensed branches, and elevated GN, for which the regulatory mechanisms are currently not clear.

Transcription factors were also major regulators of panicle development through targeting different genes and affecting their expression. *RGN1* (*REGULATOR OF GRAIN NUMBER1*) encodes an R2R3 MYB family protein, which regulates panicle architecture through activating the expression of *LOG* (*LONELY GUY*) and promote cytokinin biosynthesis (Li et al., 2022). Mutation of *RGN1* resulted in loss of lateral grains on secondary branches (Li et al., 2022). *IPA1* (*Ideal Plant Architecture 1*)/*WFP* (*WEALTHY FARMER’S PANICLE*) encodes a transcription factor OsSPL14 (SQUAMOSA promoter binding protein-like 14), which directly targets *DEP1* to promote its expression and thus increase GN (Jiao et al., 2010; Miura et al., 2010). A point mutation in *IPA1* perturbs the targeting of *OsmiR156*, resulting in reduced tiller number, increased panicle size and GN, and improved yield potential (Jiao et al., 2010). In addition, the transcription factor OsSHI1 (SHORT INTERNODES1) was suggested to physically interact with IPA1, and to represses its transcriptional activation activity (Duan et al., 2019). Another SPL family transcription factor OsSPL18 also regulates panicle architecture and GN through activating the expression of *DEP1*, while the expression of *OsSPL18* is further regulated by microRNAs *miR156k* and *miRNA529* (Yuan et al., 2019; Yan et al., 2021).

### Regulation of GN by branch differentiation of panicle

The reproductive growth of rice begins with the transition of shoot apical meristem (SAM) into inflorescence meristem (IM). Then, the branch meristem (BM) and spikelet meristem (SM) were sequentially generated from IM (Ikeda et al., 2004; Tanaka et al., 2013; Sreenivasulu et al., 2021). Determination of the meristem identity, regulation of meristem activity and phase transition are crucial factors affecting branch differentiation and GN.

Increasing the activity of IM usually results in elevated branch number and GN. The *Gn1a* (*Grain number 1a*)/*CKX2* (*Cytokinin oxidase 2*) gene is the first major QTL implicated to GN, which encodes an enzyme involved in cytokinin (CK) degradation (Ashikari et al., 2005). Down-regulation of *Gn1a* leads to the increase of CK level in the IM, thereby enhancing IM activity, branch number, and GN (Ashikari et al., 2005). *PAP2* (*PANICLE PHYTOMER 2*) encodes a MADS-box protein which belongs to the SEPALLATA (SEP) subfamily (Kobayashi et al., 2010). *PAP2* is exclusively expressed during the primary stage of panicle development, and functions in specifying the IM identity, and promote branch differentiation through controlling the expression of *TFL1*-*like* (*TERMINAL FLOWER1*-*like*) genes (Gao et al., 2010; Kobayashi et al., 2010; Liu et al., 2013; Lin et al., 2014). *LAX1* (*LAX PANICLE 1*) and *LAX2*/*GNP4* (*GRAIN NUMBER PER PANICLE 4*) jointly regulate the initiation and maintenance of BM, thereby regulating branch differentiation and GN (Oikawa and Kyozuka, 2009; Tabuchi et al., 2011; Sreenivasulu and Schnurbusch, 2012). *NOG1* (*NUMBER OF GRAINS 1*) encodes an enoyl-CoA hydratase, which is highly expressed in the BM, and positively regulates GN and grain yield (Huo et al., 2017).

The maintenance of BM identity and the phase transition from BM to SM are regulated by the well-characterized *APO1*/*APO2*-*LARGE2* module. *APO1* (*Aberrant Panicle Organization 1*)/*SCM2* (*STRONG CULM 2*) encodes an F-box-containing protein, which functions in controlling meristem cell proliferation (Ikeda-Kawakatsu et al., 2009). *APO1* can promote the expression of class-C floral homeotic genes, and suppresses the precocious transition from BM into SM, thereby positively regulate the number of branches and GN (Huang et al., 2021a). *APO2/RFL* (*Rice FLORICAULA*) gene encodes a homolog protein of *Arabidopsis* LEAFY, which interacts and cooperates with *APO1* in regulating the phase transition (Ikeda-Kawakatsu et al., 2012). *Apo1* and *apo2* mutants both showed decreased panicle size and primary branch number, which is caused by mis-regulation of floral meristem identity (Ikeda-Kawakatsu et al., 2012). *LARGE2* encodes an E3 ubiquitin ligase, which is predominantly expressed in the developing inflorescence, and negatively regulates the stability of APO1 and APO2, thereby represses the maintenance of BM activity, decrease panicle size and GN (Huang et al., 2021a). In addition, *FZP* (*FRIZZY PANICLE*) encodes an AP2/ERF family transcription factor, which negatively regulates GN through repressing the expression of *APO2*, and promote the BM to SM transition and establishment of SM identity (Komatsu et al., 2003; Bai et al., 2016). The protein abundance of FZP is further controlled by *NAL1* (*NARROW LEAF 1*), which encodes a serine and cysteine protease and interacts with FZP to promote its degradation (Huang et al., 2018). Furthermore, *TAW1* (*TAWAWA 1*) suppresses the phase transition of BM to SM, thereby prolong the branch extension, and increase the secondary branch number (Yuan et al., 2021). *RCN1* (*RICE CENTRORADIALIS 1*) and *RCN2* also negatively regulate the transition of BM to SM, and overexpression of these two genes resulted in more high order branches and increased GN (Nakagawa et al., 2002).

The genes regulating the SM activity also have the potential to increase GN (Ren et al., 2020). *FON4* (*Floral organ number 4*) controls the activity of SM (Chu et al., 2006; Ren et al., 2019), while *MFS1* (*MULTI-FLORET SPIKELET 1*) and *MFS2* are involved in the transition from SM to floral organ (Ren et al., 2013; Li et al., 2020). Mutants of these genes resulted in multi-floret spikelets and increased GN.

### Regulation of GN by phytohormone

Phytohormones are ubiquitously involved in plant growth, development, and stress responses. The biosynthesis, metabolism, and signal transduction of phytohormones have significant impact on GN, through controlling both the panicle architecture and the branch differentiation processes (Deveshwar et al., 2020).

Cytokinin (CK) is an evolutionary conserved regulator of cell division and meristem activity in plants, which play crucial roles in the floral organ development (Kieber and Schaller, 2018; Rashotte, 2021), and is recognized as a key driver of grain yield (Jameson and Song, 2016). The level of CK in the IM is positively associated with floral organ number through promoting the activity of meristem (Ashikari et al., 2005; Kurakawa et al., 2007). *LOG* (*LONELY GUY*) encodes a phosphoribohydrolase, which catalyzes the conversion of inactive CK into its active form, and its mutation resulted in reduced number of branches and decreased GN (Kurakawa et al., 2007). *An2* (*Awn-2*) also encodes a LOG family protein, which positively regulates GN through promoting CK biosynthesis (Gu et al., 2015). On the contrary, *CKX* (*cytokinin oxidase*) genes are negative regulators of GN through degradation of CK. In addition to the previously described *GN1a*/*CKX2* (Ashikari et al., 2005), some other *CKX* genes were also found to negatively regulate GN, including *CKX9* (Huang et al., 2021b) and *CXK11* (Zhang et al., 2021a). Furthermore, the expression level of the genes related to CK biosynthesis and metabolism are under the control of transcription factors and MAPK signal cascade. *DST* encodes a zinc-finger transcription factor, which negatively regulates GN through promoting the expression of *Gn1a* (Li et al., 2013), while OsMPK6 can directly phosphorylate DST and enhance its transcriptional activation activity (Guo et al., 2020b). *GSN1* (*GRAIN SIZE AND NUMBER1*)/*GLA1* (*GRAIN LENGTH AND AWN1*)/*LARGE8* encodes a MAPK phosphatase, which regulates CK metabolism through directly dephosphorylating OsMPK6, and inactivating the MAPK signal (Guo et al., 2018; Xu et al., 2018; Wang et al., 2019; Zhang et al., 2019a). *ERECTA1* (*OsER1*) acts upstream of *GSN1*, and negatively regulates GN through promoting CK metabolism (Guo et al., 2020a,b). Moreover, the mediator protein OsMED25 physically interacts with DST and functions as a coactivator through recruiting RNA polymerase II to the promoter of *OsCKX2* and promote its transcription (Lin et al., 2022). These genes coordinately regulate panicle architecture and GN through integrative control of CK homeostasis.

Gibberellin (GA) is known as a positive regulator of cell division and elongation in vegetative organs (Binenbaum et al., 2018). However, GA play negative roles in regulating the IM activity (Kwon and Paek, 2016; Su et al., 2021). Previous studies have shown that the *OsCYP71D8L* (*CYTOCHROME P450-71 D8L*) gene negatively regulates GN and panicle length through GA biosynthesis (Gao and Chu, 2020; Zhou et al., 2020). GA signal transduction depends on the *GID* family genes. It was suggested that *Sped1-D* can repress the expression of several *GID1L2* genes, and promote the elongation of pedicels and secondary branches, thereby increase GN (Jiang et al., 2014). Furthermore, a crosstalk between GA and cytokinin was implicated in GN regulation. *GNP1* (*grain number per panicle1*) encodes a GA20 oxidase, and increased expression of *GNP1* caused a feed-back regulation of GA catabolism genes, reduced GA accumulation and enhanced CK level in panicle meristems to increase GN (Bessho-Uehara et al., 2016).

Auxin is recognized as a negative regulator of IM activity (He et al., 2018; Goetz et al., 2021). The dynamic efflux of auxin conducted by PIN (PIN-FORMED) protein family are essential for the establishment of axillary meristems (Deveshwar et al., 2020). *PAY1* (*PLANT ARCHITECTURE AND YIELD1*) improves GN and plant architecture through influencing polar auxin transport and shifting auxin distribution (Zhao et al., 2015). *NAL1* also functions in the polar transport of auxin, and overexpressing *NAL1* can promote panicle branching and GN (Qi et al., 2008).

Brassinosteroid (BR) is an essential regulator of cell expansion and grain size (Li et al., 2018a; Fan and Li, 2019). It is revealed that BR signal is also involved in the meristem differentiation during panicle development. The QTLs *CPB1* (*CLUSTERED PRIMARY BRANCH1*), *GNS4* (*grain number and size on chromosome 4*) and *PMM1* (*Panicle Morphology Mutant1*), were independently identified to regulate GN and grain size through affecting spikelet meristem differentiation as well as panicle architecture (Wu et al., 2016; Zhou et al., 2017; Li et al., 2018c). Further analysis identified them as multiple alleles of *D11* (*DWARF11*), a cytochrome P450 encoding gene involved in BR biosynthesis pathway (Tanabe et al., 2005). In addition, it has been revealed that ABA and ethylene negatively regulate GN (Hirose et al., 2007; Wuriyanghan et al., 2009). It is worth noting that GN is a complex agronomic trait, and its regulation is usually the result of the synergistic effect of multiple phytohormones. Dissecting the genetic architecture and molecular mechanism of these genes would facilitate the genetic improvement of grain production in rice and other crops.

### Regulation of GN by vascular development

The vascular system connects the entire plant body and conducts the long-distance transport of water, inorganic salts, nutrients and assimilates, which are crucial for plant growth and grain yield (De Rybel et al., 2016; Agusti and Blazquez, 2020). The vascular bundles in the stem internode consists of the large vascular bundles (lvbs) arranged in the inner side of the cortex, and the small vascular bundles (svbs) arranged around the outer side of stem. Each vascular bundle consists of the phloem and the xylem. The vascular bundles in panicle neck determines the transport efficiency of photoassimilates from “source” leaf to “sink” grain. Furthermore, the lvbs in panicle neck are directly connected to the primary branches of the panicle (Liao et al., 2021). Therefore, the number of lvbs in the panicle neck is positively correlated with the number of branches and GN (Zhai et al., 2018; Fei et al., 2019; Liao et al., 2021).

Emerging evidence have revealed an association between vascular development and GN. *Ghd7* gene is highly expressed in the vascular tissue, and its elite allele shows an improved vascular system and increased GN (Xue et al., 2008). Genome-wide association analysis also revealed that *Ghd7* is a key locus affecting vascular development (Liao et al., 2021). The *NAL1* (*Narrow Leaf 1*) gene, which is associated with leaf development, has a significant effect on GN in both *indica* and *japonica* populations (Chen et al., 2012; Wang et al., 2020c). Recent research shows that *NAL1* can also significantly affect the vascular bundle morphology in leaves and panicle neck (Liao et al., 2021). The *qPE9-1/DEP1* and *EP2* genes associated with erect panicle shape were also found to regulate the patterning and development of large vascular bundles in the panicle neck (Zhu et al., 2010; Xu et al., 2015b). *APO1* is predominantly expressed in developing vascular tissues, and promotes the translocation of photoassimilates (Terao et al., 2010). *SP1* is also expressed in the vascular bundle of developing panicles, where it functions as a nitrate transporter (Li et al., 2009).

Studies on vascular patterning and development revealed key genes which possess the potential to increase GN. The transaldolase gene *TAL* is a key regulator of vascular development in rice, which also has a positive effect on GN and grain yield (Yang et al., 2015). Knock down of *OsTAL* reduced the number and area of stem lvbs, and significantly decreased GN and grain yield (Yang et al., 2015). The *OsCOMT* (*caffeic acid O-methyl transferase*) gene, which encodes the rate-limiting enzyme in melatonin biosynthesis, positively regulates GN and grain yield through promoting vascular development and delaying leaf senescence (Huangfu et al., 2022). Overexpression of *OsCOMT* can significantly improve the vascular bundle size and number, and increase GN (Huangfu et al., 2022). It is suggested that the improved vascular system (flow) may promote the translocation of photoassimilates from source to sink organs (Li et al., 2018b). These observations also suggested that the vascular development associated genes may be useful in improving GN and rice grain yield.

### Pleiotropy of the genes regulating GN

Many genes associated with GN have been revealed to possess pleiotropic effects in other important agronomic traits, such as tiller number, grain shape, grain weight, heading date and plant architecture. Tillering and panicle branching are both controlled by the activity of axillary meristem (Liang et al., 2014). Therefore, the genes involved in axillary meristem establishment and maintenance often exhibit co-regulation of GN and tiller number. *MOC1* (*MONOCULM 1*)/*GNP6* (*grain number per panicle 6*) encodes a GRAS (GAI, RGA and SCR) family protein, which is an essential regulator for the initiation of axillary meristem, and its null mutation resulted in almost complete loss of tillers and arrested branch growth (Li et al., 2003; Shao et al., 2019). *LAX1*, *LAX2*, *APO1* and *APO2* were required for the maintenance of axillary meristems (Oikawa and Kyozuka, 2009; Tabuchi et al., 2011), therefore, mutation of these genes also significantly reduced tiller number and GN (Zhang et al., 2021b). However, GN and tiller number can sometimes show opposite regulations. *PAY1* encodes a peptidase S64 domain protein, which is associated with auxin transport (Zhao et al., 2015). Enhanced expression of *PAY1* increases the number of secondary branches, but reduces the number of tillers (Zhao et al., 2015). Moreover, *IPA1* also increases GN but reduces PN through directly binding to the promoter of *TB1* (*Teosinte Branched 1*) and *DEP1* to promote their transcription, thereby suppress rice tillering, promote branching, and regulate rice plant architecture (Miura et al., 2010; Lu et al., 2013).

The regulation of GN and grain size (GS) or grain weight (GW) often show an antagonistic relationship (Fan and Li, 2019). Down-regulation of *GS3* (*GRAIN SIZE/SHAPE 3*; Fan et al., 2006; Mao et al., 2010), *GSN1* (*GRAIN SIZE AND NUMBER1*; Zhang et al., 2019a), and *GW2* (*GRAIN WIDTH 2*; Song et al., 2007) resulted in increased GW but reduced GN; while down-regulation of *GW10* (*GRAIN WIDTH 10*) decreased GW but increased GN (Zhan et al., 2021). On the other hand, down-regulation of GN-associated genes *DEP1* (Huang et al., 2009b; Yi et al., 2011; Li et al., 2019), *GAD1* (*grain number, grain length and awn development 1*; Bessho-Uehara et al., 2016; Jin et al., 2016), and *FZP* (Bai et al., 2016; Fujishiro et al., 2018) increased GN but reduced GW. This antagonistic relationship between GN and GW can be attributed to the competition effects for photoassimilates (Fan and Li, 2019). The balancing between GW and GN would be an important target in the future improvement of rice gain yield.

Some genes regulating heading date were also found to be associated with GN. *Ghd7* (*Grain Number, Plant Height, and Heading Date 7*) gene encodes a CCT domain protein, and its overexpression under long-day condition delays the heading date and increased GN (Weng et al., 2014). *Ghd8* gene can simultaneously regulate the heading date, tiller number, plant height and the number of branches, thereby affect GN (Yan et al., 2011). Such synergistic regulation of GN and heading date has also been observed in studies on other genes including *APO2*/*RFL* (Ikeda-Kawakatsu et al., 2012), *RCN1* (*RICE CENTRORADIALIS 1*; Nakagawa et al., 2002; Wang et al., 2020b), *RCN2* (Nakagawa et al., 2002), and *OsCOL13* (*CONSTANS-LIKE 13*; Sheng et al., 2016). However, the regulatory mechanism of the association between GN and heading date remains elusive.

It was suggested that GN-associated genes can also affect other important agronomic traits, such as nutrient metabolism, plant architecture, and stress response. For instance, *qPE9-1/DEP1* has been revealed as a multifunctional regulator of nitrogen use efficiency (NUE; Dong et al., 2022), root elongation and phosphorus uptake (Wang et al., 2021), as well as drought stress response (Zhang et al., 2015). In addition, *DEP1* can interact with *RGA1* (*Rice G-protein Alpha subunit1*) and *RGB1* (*Rice G-protein Beta subunit1*) to increase nitrogen absorption and utilization, and ultimately increase the plant biomass and grain yield (Sun et al., 2014). *OsEBS* (*ENHANCING BIOMASS AND SPIKELET NUMBER*) positively regulates the plant height, leaf size and biomass in addition to GN (Dong et al., 2013). *DST* functions in rice drought and salt tolerance through modulating stomatal aperture, and also positively regulates GN (Huang et al., 2009a). Moreover, loss of function of *Gn1a*/*OsCKX2* not only increased GN, but also enhanced lodging resistance through accelerating root development and increasing the culm diameter (Tu et al., 2022). Further studies would be expected for elucidating the relationship between GN and these traits, and revealing the application value of these pleiotropic genes in rice breeding.

GN is a common domestication syndrome trait in cereal crops. Interestingly, many GN-associated genes were involved in the regulation of other domestication related traits. *PROG1* (*PROSTRATE GROWTH 1*) is a key gene in the process of rice domestication, which controls the critical transition from prostrate to erect growth, and changes the plant architecture (Tan et al., 2008; Huang et al., 2020). *PROG1* is predominantly expressed in the axillary meristems, and promotes GN through increasing the number of primary and secondary branches (Jin et al., 2008). *GAD1*/*RAE2* (*regulator of awn elongation2*)/ *GLA* (*Grain Length and Awn Development*) encodes a secreted peptide, and the loss-of-function of *GAD1* resulted in decreased grain length, increased GN, and loss of awn, suggestive important role of *GAD1* in the domestication of rice (Bessho-Uehara et al., 2016; Jin et al., 2016; Zhang et al., 2019b; Xu and Sun, 2021). The mutants of *An-1* (*Awn-1*) and *An-2* showed reduced awn length, increased GN and GW, and these genes have experienced artificial selection during rice domestication (Luo et al., 2013; Gu et al., 2015). Furthermore, recent study revealed that *OsKRN2* (*Kernel Row Number 2*), which negatively regulates GN, has undergone convergent selection with its maize ortholog *KRN2* during the domestication of rice and maize (Chen et al., 2022). These findings partially revealed the genetic basis and molecular mechanisms underlying the selective forces of GN and other domestication related traits. Understanding the molecular mechanism of the co-regulation of these traits would provide novel insights for the improvement of grain yield in crop genetic improvement.

### Conclusions and future perspectives

**Figure 1:**
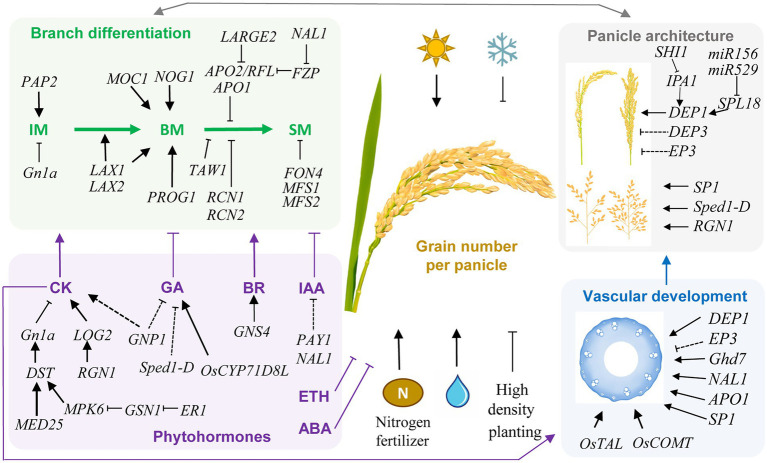
Schematic representation of genetic and environmental factors controlling grain number per panicle (GN) in rice. Key genes and regulatory pathways in controlling GN are indicated. Arrows indicate positive regulation, while T lines indicate negative regulation. Dashed lines indicate indirect regulations. Rice GN is determined by the panicle architecture and branch differentiation, which are associated with phytohormones and vascular development. These regulatory pathways are interconnected in regulating GN. In the determination of panicle architecture, the *DEP1*, *DEP3*, and *EP3* genes control the dense and erect panicle type, while *SP1*, *Sped1-D*, and *RGN1* regulate panicle length and branch number. Transcription factors IPA1 and SPL18 positively regulates the expression of *DEP1*. SHI1 interacts with IPA1 and inhibit its activity, while microRNAs *miR156* and *miR529* regulate the expression level of *SPL18*. The branch differentiation involves the activity and phase transition of inflorescence meristem (IM), branch meristem (BM), and spikelet meristem (SM). Increasing the meristem activity and delaying the transition from BM to SM have a positive effect on GN. Phytohormones were also crucial regulators of GN through manipulating branch differentiation, among which cytokinin (CK) and brassinosteroid (BR) are positive regulators, while indole 3-acetic acid (IAA), gibberellin (GA), abscisic acid (ABA), and ethylene (ETH) are negative regulators. The key GN regulator *GN1a* encodes a CKX, which negatively regulate CK level, and is regulated by the *ER1*-*GSN1*-*MPK6*-*DST* signal cascade, while MED25 functions as a coactivator with DST to promote expression of *GN1a*. Vascular development is closely associated with GN through affecting the panicle architecture and translocation capability for water and nutrients. Many GN-associated genes also function in regulating vascular development, while the key vascular development regulators *OsTAL* and *OsCOMT* both have significant positive effects on GN. Moreover, vascular development is positively regulated by CK. In addition, light, water, and nitrogen availability positively regulate GN, while low temperature and high planting density negatively control GN. These genetic and non-genetic factors together determine the GN of rice.

During the past two decades, substantial progresses have been made in understanding the genetic and molecular factors in determining GN in rice (Figure 1; Table 1). GN is mainly determined by panicle architecture and branch differentiation. Panicle architecture consists of the panicle length and the number and arrangement of rachis branches, while branch differentiation is controlled by the establishment, maintaining, phase transition, and differentiation of IM, BM, and SM. These processes are regulated by various phytohormones and G-protein signal pathways, and are closely associated with the vascular development. Furthermore, the GN-associated genes play pleiotropic roles in regulating PN, GW, flowering time and domestication related traits. However, rice GN is a complex quantitative trait which is regulated by multiple factors. In addition to these major aspects, some other factors, including spikelet sterility (Heng et al., 2018; Sekhar et al., 2021), nitrogen allocation (Guo et al., 2020a), sugar transport (Seki et al., 2015; Xu et al., 2019), and circadian clock regulation (Wang et al., 2020a), might also participate in the regulation of rice GN.

**Table 1:** List of the genes involved in rice grain number per panicle regulation.

Gene name	Locus	Protein	Function	References
*APO1*	LOC_Os06g45460	F-box protein	Control meristem cell proliferation; enhance the formation of vascular bundle systems	Ikeda-Kawakatsu et al. (2009), Terao et al., (2010)
*APO2*/*RFL*	LOC_Os04g51000	Transcription factor RFL	Control inflorescence and flower development	Kyozuka et al. (1998), Ikeda-Kawakatsu et al. (2012)
*COMT*	LOC_Os08g06100	Caffeic acid O-methyltransferase	Promote GN through regulating vascular development	Huangfu et al. (2022)
*DEP1/qPE9-1*	LOC_Os09g26999	G-protein gamma subunit	Regulate GN, panicle length, and grain weight	Huang et al. (2009b), Zhou et al. (2009), Sun et al. (2014), Xu et al. (2015b)
*DEP2*	LOC_Os07g42410	Unknown plant-specific protein	Control panicle outgrowth and elongation	Li et al. (2010)
*DEP3*	LOC_Os06g46350	Patatin-related phospholipase A	Regulate the formation of vascular bundles	Qiao et al. (2011)
*DST*	LOC_Os03g57240	Zinc-finger transcription factor	Directly regulate *OsCKX2* expression in the reproductive meristem	Li et al. (2013)
*EBS*	LOC_Os05g51360	Similar to the N-terminal conserved ATPase domain of Hsp70	Enhancing biomass and spikelet number	Dong et al. (2013)
*EP3*	LOC_Os02g15950	F-box protein	Regulates panicle architecture and vascular development	Piao et al. (2009)
*ER1*	LOC_Os06g10230	receptor-like protein kinase	Regulate CK metabolism through the MAPK signal cascade	Guo et al. (2020b)
*FON4*	LOC_Os11g38270	Receptor-like protein kinase	Prevents the multi-floret spikelet through controlling SM identity	Ren et al. (2019)
*FZP*	LOC_Os07g47330	ERF transcription factor	Promote GN through establishing floral organ identity	Komatsu et al. (2003), Bai et al. (2016)
*GAD1*/*RAE2*	LOC_Os08g37890	Cysteine-rich secretory peptide	Regulates GN, grain length, and awn development	Bessho-Uehara et al. (2016), Jin et al. (2016)
*Gn1a*/*CKX2*	LOC_Os01g10110	Cytokinin oxidase CKX2	Reduce GN through cytokinin metabolism	Ashikari et al. (2005), Gouda et al. (2020a)
*Ghd7*	LOC_Os07g15770	CCT (CO, CO-LIKE and TIMING OF CAB1) domain protein	Regulates grain number, plant height, and heading date; promote vascular development	Xue et al. (2008), Weng et al. (2014)
*Ghd7.1*	LOC_Os07g49460	pseudo-response regulator (PRR) protein	Delays rice heading and enhances grain productivity	Luo et al. (2013), Yan et al. (2013)
*Ghd8*	LOC_Os08g07740	HAP3 subunit of the HAP	Regulate grain number, plant height, and heading date	Yan et al. (2011)
*GNP1*	LOC_Os03g63970	Gibberellin biosynthesis enzyme GA20ox1	Promote gibberellin biosynthesis	Bessho-Uehara et al. (2016)
*GNS4*	LOC_Os04g39430	Cytochrome P450 protein	Positively regulate GN and GS through the BR pathway	Zhou et al. (2017)
*GSN1*/*GLA1*/ *LARGE8*	LOC_Os05g02500	MAPK phosphatase	Regulate CK metabolism through inactivating MAPK signal cascade	Guo et al. (2018), Xu et al. (2018), Wang et al. (2019), Zhang et al. (2019a)
*IPA1*/*OsSPL14*	LOC_Os08g39890	SOUAMOSA PROMOTER BINDING PROTEIN-LIKE transcription factor	Promote shoot branching through transcriptional activation of *DEP1*	Jiao et al. (2010), Miura et al. (2010), Lu et al. (2013)
*LARGE2*	LOC_Os12g24080	HECT-domain E3 ubiquitin ligase	Negatively regulate GN through affecting the stability of APO1 and APO2	Huang et al. (2021a)
*LAX1*	LOC_Os01g61480	bHLH transcription factor	Regulate axillary meristems formation	Oikawa and Kyozuka (2009)
*LAX2*/*GNP4*	LOC_Os04g32510	Nuclear protein with a plant-specific conserved domain	Interact with LAX1; regulate axillary meristem formation and lateral branching	Tabuchi et al. (2011), Zhang et al. (2018)
*MED25*	LOC_Os09g13610	Mediator protein	Interact with DST to promote expression of *Gn1a*	Lin et al. (2022)
*MFS1*	LOC_Os05g41760	AP2 domain containing protein	Repress SM determinacy and floral organ identity	Ren et al. (2013)
*MFS2*	LOC_Os04g47890	MYB transcription factor	Repress SM determinacy and floral organ identity	Li et al. (2020)
*MOC1*/*GNP6*	LOC_Os06g40780	GRAS-family nuclear protein	Promote axillary meristem initiation	Zhang et al. (2021b)
*NAL1*/*qFLW4*	LOC_Os04g52479	Trypsin-like serine and cysteine protease	Promote degradation of FZP; positively regulate leaf and vascular development	Qi et al. (2008), Fujita et al. (2013), Xu et al. (2015a), Huang et al. (2018), Lin et al. (2019), Wang et al. (2020c)
*NOG1*	LOC_Os01g54860	Enoyl-CoA hydratase/isomerase	Promote GN without affecting other yield traits	Huo et al. (2017)
*PAP2*/*MADS34*	LOC_Os03g54170	SEP-like MADS box transcription factor	Positively control spikelet meristem identity	Gao et al. (2010), Kobayashi et al. (2010), Lin et al. (2014)
*PAY1*	LOC_Os08g31470	Trypsin-like serine and cysteine protease	Improve plant architecture through affecting polar auxin transport and endogenous IAA distribution	Zhao et al. (2015)
*PROG1*	LOC_Os07g05900	Cys2-His2 zinc-finger protein	Regulate erect growth, promote GN and grain yield	Tan et al. (2008)
*RCN1*	LOC_Os03g17350	White-brown complex homolog protein	Promote branching through delaying the phase transition	Nakagawa et al. (2002)
*RCN2*	LOC_Os02g32950	Phosphatidylethanolamine-binding protein	Promote branching through delaying the phase transition	Nakagawa et al. (2002)
*RGN1*	LOC_Os01g49160	R2R3 MYB transcription factor	Promote GN through regulating *LOG* expression	Li et al. (2022)
*RLB*	LOC_Os07g03770	KNOX type homebox protein	Promote GN through epigenetic silencing of *OsCKX4*	Wang et al. (2022a)
*SH1*	LOC_Os09g36160	Transcription factor	Interacts with IPA1 and represses the transcriptional activation ability	Duan et al. (2019)
*SP1*	LOC_Os11g12740	Putative peptide transporter (PTR) family protein	Regulate panicle architecture through nitrate transport	Li et al. (2009)
*SPL18*	LOC_Os09g32944	SOUAMOSA PROMOTER BINDING PROTEIN-LIKE transcription factor	Promote expression of *DEP1*	Yuan et al. (2019)
*sped1*-*D*	LOC_Os06g39650	Pentatricopeptide repeat protein	Prompt the shortening of pedicels and secondary branches through repressing the GA signal transduction	Jiang et al. (2014)
*TAL*	LOC_Os01g70170	Transaldolase	Promote vascular development	Yang et al. (2015)
*TAW1*	LOC_Os10g33780	Unknown nuclear protein	Promote panicle development	Yuan et al. (2021)

Identification of GN-associated genes are of vital importance both for understanding the regulatory network of GN and for the improvement of rice yield. In addition to the traditional genetic mapping approach, GWAS (genome-wide association studies) also provides an effective tool in unraveling the genetic basis of GN and other yield traits (Rebolledo et al., 2016; Xiao et al., 2017). Moreover, the advent of third-generation long-range genome sequencing and pangenomes have greatly enriched the genomic information and expanded genetic diversity of rice and other crops (Zhao et al., 2018; Alonge et al., 2020). Crop pangenome studies highlighted structural variants and their association with important agronomic traits (Gabur et al., 2019), which would have great potential in GN-associated gene mining and yield improvement. On the other hand, innovation of rice germplasm populations with rich genetic and phenotypic variations are essential for mining novel genes and QTLs. Construction of multi parent populations (MPPs), including MAGIC (multiparent advanced generation inter-cross) and MCC-NAM (mini-core collection nested association mapping) populations, provide effective tools for the identification of novel genes controlling complex traits (Scott et al., 2020). Compared with traditional bi-parent populations, MPPs effectively expanded genetic diversity and increased genetic recombination. The application of these MPPs provide higher mapping power and resolution in exploring genetic architecture of yield traits in rice (Zaw et al., 2019; Han et al., 2020; Ayaad et al., 2021; Huerta et al., 2021), and would be effective strategies in the cloning of GN-associated genes and rice breeding. In addition, current studies also shed light on the roles of epigenetic modification related genes in GN regulation (Zhang et al., 2017; Wang et al., 2022a). Despite the increasing attention on the importance of epigenetic regulations in plant growth, stress response, and crop yield (Lu et al., 2018, 2020), the significance and mechanisms of epigenetic modification related genes in GN regulation remains largely elusive. Mining the epigenetic genes and mechanisms underlying GN regulation through bisulfite sequencing, chromatin immunoprecipitation (ChIP) assay, and epigenome editing based on RNA-dependent DNA methylation (Wakasa et al., 2018), will provide novel insight into the regulations on GN. The identification and mechanic revelation of genes controlling GN will further guide the molecular design breeding of rice.

The application of GN-associated genes in rice breeding is a pivotal task in the future genetic improvement. Gene editing technologies, including transcription activator-like effector nucleases (TALENs), zinc-finger nucleases (ZFNs), and clustered regularly interspaced short palindromic repeats (CRISPR-Cas9) system, are promising tools for reshaping crop breeding. These technologies enabled flexibility in improving target traits through precise targeting of multiple genes (Zhu et al., 2020; Ganie et al., 2021; Lu et al., 2021; Mohd Saad et al., 2022). It is suggested that CRISPR-Cas9 editing of GN-associated genes, including *GN1a*, *DEP1*, and *IPA1*, can significantly increase GN and rice grain yield (Li et al., 2016, 2021a). Therefore, editing of GN-associated genes in elite cultivars through these approaches would be effective and promising to accelerate the utilization of these genes in the breeding process. Genomic selection (GS) estimates the effects of all markers in a training population, and use this information to predict the breeding value of genotyped individuals (Crossa et al., 2017; Xu et al., 2020). GS holds enormous potential in transferring the elite allele into breeding cultivars and accelerating the breeding process (Xu et al., 2021b), which has been successfully applied in rice breeding (Cui et al., 2020; Xiao et al., 2021). Furthermore, the development of machine learning, deep learning, and neural network strategies, has greatly improved the efficiency in phenotyping and analyzing environmental variables that affect phenotypes. These strategies, combined with high throughput plant phenotyping technique, provide efficient and effective solution for improving the predictive capability and trait improvement (Bayer and Edwards, 2021). Moreover, the recent developed multi-trait GS technology offers a powerful and efficient solution for improving the predictive ability for complex traits (Wang et al., 2017; Moeinizade et al., 2020; Xu et al., 2021a). Considering the pleiotropy of GN-associated genes, multi-trait GS would be favorable for the improvement of GN and other associated traits in rice breeding, such as GW, plant architecture, and improved vascular system. The integration of gene identification and molecular breeding strategies will benefit for the future improvement of GN and other agronomic traits in rice.

## Author contributions

YL, ZY, and CX conceived the idea and wrote the manuscript. YL, MC, HW, RC, and TT collected the materials. YZ, YX, and PL prepared the figures. YZ and YY revised the manuscript. All authors contributed to the article and approved the submitted version.

## Funding

This work was supported by grants from the National Natural Science Foundation of China (32100448, 32070558, 32061143030, 32170636, and 31970248), the Priority Academic Program Development of Jiangsu Higher Education Institutions (PAPD), the Natural Science Foundation of Jiangsu Province (BK20210799), the Seed Industry Revitalization Project of Jiangsu Province [JBGS(2021)009], and the Project of Hainan Yazhou Bay Seed Laboratory (B21HJ0223).

## Conflict of interest

The authors declare that the research was conducted in the absence of any commercial or financial relationships that could be construed as a potential conflict of interest.

## Publisher’s note

All claims expressed in this article are solely those of the authors and do not necessarily represent those of their affiliated organizations, or those of the publisher, the editors and the reviewers. Any product that may be evaluated in this article, or claim that may be made by its manufacturer, is not guaranteed or endorsed by the publisher.
